# Soft Tissue Infection of Immunocompetent Man with Cat-Derived *Globicatella* Species

**DOI:** 10.3201/eid2908.221770

**Published:** 2023-08

**Authors:** Nick K. Jones, Juliana Coelho, Julie M.J. Logan, Karen Broughton, Katie L. Hopkins, Bruno Pichon, Isabelle Potterill, Yu Wan, Alex W.N. Reid, Theodore Gouliouris

**Affiliations:** Cambridge University Hospitals, Cambridge, UK (N.K. Jones, A.W.N. Reid, T. Gouliouris);; University of Cambridge, Cambridge (N.K. Jones, T. Gouliouris);; United Kingdom Health Security Agency Antimicrobial Resistance and Healthcare Associated Infections Reference Unit, Colindale, UK (J. Coelho, J.M.J. Logan, K. Broughton, K.L. Hopkins, B. Pichon, I. Potterill, Y. Wan);; Imperial College London, London, UK (K.L. Hopkins, B. Pichon, Y. Wan)

**Keywords:** soft tissue infection, immunocompetent man, bacteria, *Globicatella sanguinis*, *Globicatella sulfidifaciens*, bites, cat-derived, cat, zoonoses, United Kingdom

## Abstract

We report a novel *Globicatella* species causing extensive soft tissue infection in a man bitten by a stray domestic cat in the United Kingdom. We identified this bacterium by 16S rRNA gene sequencing, whole-genome sequencing, and biochemical profiling and determined antimicrobial drug susceptibility.

Cats are major reservoirs of zoonotic infections. Their long, sharp teeth predispose to deep-tissue bite injuries, and direct inoculation of feline saliva gives high risk for secondary infection. Infecting pathogens usually reflect colonizing oral microbiota; *Pasteurella* and *Streptococcus* species are the most common (*1*). Bacteria of the genus *Globicatella* are small, gram-positive cocci that resemble viridans-group streptococci. *Globicatella sanguinis* is the only known species to cause human infection, having been implicated in small numbers of bloodstream, heart, central nervous system and urinary tract infections (*2*). *G. sulfidifaciens* is the only other known *Globicatella* species, but human infection has not been described (*3*). We report a novel *Globicatella* species causing extensive soft tissue infection and tenosynovitis in an immunocompetent man after cat bite injuries.

A 48-year-old obese man came to the emergency department in 2020 because of painful bilateral hand swelling, 8 hours after sustaining several bites from a single feral cat. He had multiple puncture wounds and abrasions, without evidence of surrounding cellulitis. His wounds were bathed in povidone‒iodine solution and dressed, and a booster dose of tetanus vaccine was administered. He was discharged and given oral doxycycline, ciprofloxacin, and metronidazole treatment because of history of penicillin allergy. He returned to the emergency department 24 hours later because of evolving flexor sheath infection in his left little and right middle fingers and cellulitis of both forearms (Figure). He was given intravenous vancomycin, ciprofloxacin, and metronidazole, then underwent debridement and washout. He was given 5 days of oral doxycycline and metronidazole postoperatively and made a full recovery. The patient provided fully informed, written consent for this case to be published, with accompanying clinical photographs.

**Figure:**
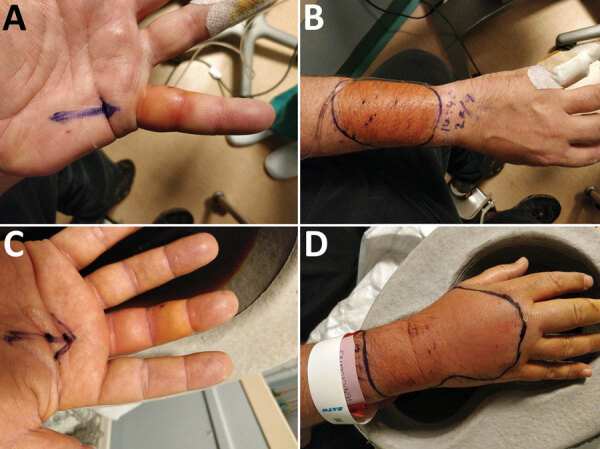
Clinically apparent areas of infection with *Globicatella* species in patient with soft tissue ianfection after cat bite, United Kingdom: A) left little finger, B) right forearm, C) right middle finger, and D) right hand.

 We obtained tissue samples from debridement sites and a swab specimen from the right middle finger for microbiological analysis with Gram stain and bacterial culture on blood, chocolate, cystine-lactose-electrolyte—deficient, and fastidious anaerobe agars. No organisms were seen on Gram stain. A scant growth of *Staphylococcus epidermidis* was isolated from the right middle finger tissue sample, as well as a *Streptococcus*-like organism that grew best on chocolate agar. Culture yield may have been affected by previous antimicrobial drug treatment. Matrix-assisted laser desorption/ionization time-of-flight mass spectrometry (Bruker, https://www.bruker.com) of the *Streptococcus*-like organism gave no reliable identification (score 1.31). We referred this isolate (designated G1610988) to the UK Health Security Agency Reference Laboratory for further characterization.

We obtained partial 16S rRNA gene sequence data after block-based PCR as described (*4*). Those data did not match any named species in the GenBank database. The closest sequence database match was to *Globicatella* sp. feline oral taxon 122 (99%–100% identity) (*5*).

API Rapid ID 32 Strep analysis (bioMérieux, https://www.biomerieux.com) gave an organism identification of *Erysipelothrix rhusiopathiae* (98.7%). The isolate was negative for pyrrolidonyl aminopeptidase and leucine aminopeptidase and positive for bile aesculin (Diatabs; Rosco Diagnostica, https://www.rosco.dk) diagnostic tablets for bacterial identification. Repeat matrix-assisted laser desorption/ionization time-of-flight mass spectrometry at the reference laboratory gave no reliable identification (score 1.41). Comparison with biochemical profiling of *G. sanguinis* and *G. sulfidifaciens* type strains showed notable differences (Appendix). The conditions required for culture were not different between *Globicatella* species.

We conducted antimicrobial drug susceptibility testing by using MIC gradient strips (Liofilchem, https://www.liofilchem.com) and PK/PD and non–species-related breakpoints (*6*). Gentamicin was identified as an unsuitable treatment option, MIC 1.0 mg/L (breakpoint 0.5 mg/L). Treatments suitable for use with caution were ampicillin, MIC ≤0.016 mg/L (2.0 mg/L); cefotaxime, MIC 0.004 mg/L (1.0 mg/L); penicillin, MIC ≤0.016 mg/L (0.25 mg/L); linezolid, MIC 1.0 mg/L (2.0 mg/L); ciprofloxacin, MIC 0.032 mg/L (0.25 mg/L); and moxifloxacin, MIC 0.016 mg/L (0.25 mg/L). No PK/PD non–species-related breakpoints were available for teicoplanin, MIC 0.032 mg/L; vancomycin, MIC 0.25 mg/L; clindamycin, MIC 1.0 mg/L; erythromycin. MIC 0.032 mg/L; tetracycline, MIC 0.064 mg/L; chloramphenicol, MIC 2.0 mg/L; or rifampin, MIC 0.004 mg/L.

To corroborate the 16S rRNA gene sequence results, we conducted whole-genome sequencing on a HiSeq 2500 platform (Illumina, https://www.illumina.com) at the UK Health Security Agency Central Sequencing Laboratory by using its standard paired-end 101-bp sequencing protocol. We extracted genomic DNA from lysate by using the QIAsymphony DSP DNA Mini Kit and automated QIAsymphony SP/AS Instruments (QIAGEN, https://www.qiagen.com). We trimmed and filtered sequencing reads by using Trimmomatic (*7*) for quality control, then assembled by using SPAdes version 3.15 (*8*). Comparison with published *Globicatella* genomes by using FastANI (*9*) showed an average nucleotide divergence of 20.29% to its most closely related cluster (*G. sulfidifaciens*), suggesting a distinct and previously undescribed species (Appendix Figure).

Genomic sequences of isolate G1610988 have been deposited in the European Nucleotide Archive (Biosample accession no. SAMEA110751862). Partial sequence of the 16S rRNA gene has been deposited in GenBank (accession no. MW242777).

In conclusion, cat bites are common sources of zoonotic infection. This report highlights the role of cats as reservoirs of as yet undiscovered bacterial species that have human pathogenic potential. Currently recommended antimicrobial drug regimens for treating cat bites can be expected to include the *Globicatella* species described.

Appendix Additional information on soft tissue infection of immunocompetent man with cat-derived *Globicatella* species.
