# Supplementary material for: Soft Tissue Infection of Immunocompetent Man with Cat-Derived Globicatella Species
Source: Emerg Infect Dis. 2023 Aug;29(8):1684–7. doi: 10.3201/eid2908.221770 (PMC10370850; doi:10.3201/eid2908.221770)
Supplement: Appendix — Additional information on soft tissue infection of immunocompetent man with cat-derived Globicatella species. [file 22-1770-Techapp-s1.pdf]

Article DOI: https://doi.org/10.3201/eid2908.221770
EID cannot ensure accessibility for Supplemental Materials supplied by authors. Readers who have difficulty accessing supplementary content should contact the authors for assistance.

Soft Tissue Infection of Immunocompetent Man with Cat-Derived *Globicatella* Species

Appendix

Appendix Table. Biochemical profiling and antimicrobial susceptibility

Test	Novel *Globicatella* isolate G1610988	*Globicatella sanguinis* Type strain*	*Globicatella sulfidifaciens* Type strain†
Component test of the API RAPID ID 32 STREP assay			
ADH	Negative	Negative	Negative
BGLU	Negative	Positive	Positive
BGAR	Positive	Positive	Positive
BGUR	Negative	Negative	Positive
AGAL	Negative	Negative	Positive
PAL	Negative	Negative	Negative
RIB	Negative	Negative	Negative
MAN	Negative	Positive	Negative
SOR	Negative	Negative	Negative
LAC	Negative	Negative	Positive
TRE	Negative	Positive	Positive
RAF	Negative	Positive	Positive
VP	Negative	Negative	Negative
APPA	Negative	Positive	Positive
BGAL	Negative	Positive	Positive
PYRA	Positive	Negative	Positive
BNAG	Negative	Negative	Negative
GTA	Negative	Negative	Negative
HIP	Negative	Positive	Positive
GLYG	Negative	Positive	Positive
PUL	Negative	Negative	Positive
MAL	Negative	Positive	Positive
MEL	Negative	Positive	Positive
MLZ	Negative	Negative	Negative
SAC	Negative	Positive	Positive
LARA	Negative	Negative	Negative
DARL	Negative	Negative	Negative
MBDG	Negative	Negative	Positive
TAG	Negative	Negative	Negative

Test	Novel *Globicatella* isolate G1610988	*Globicatella sanguinis* Type strain*	*Globicatella sulfidifaciens* Type strain†
BMAN	Negative	Negative	Negative
CDEX	Negative	Negative	Negative
URE	Negative	Negative	Negative
Overall	Good identification *Erysipelothrix rhusiopathiae* (98.7%)	Very good identification *G. sanguinis* (99.9%)	Very good identification *G. sanguinis* (99.7%)
Other tests			
Pyrrolidonyl aminopeptidase	Negative *positive by API	Positive	Positive
Leucine aminopeptidase	Negative	Negative	Negative
Bile aesculin	Positive	Positive	Positive
Initial MALDI-TOF MS	No reliable identification (score 1.31)	N/A	N/A
Repeat MALDI-TOF MS (reference laboratory)	No reliable identification (score 1.41)	*G. sanguinis* (score 2.54)	*G. sanguinis* (score 2.36)

Antibiotic susceptibility	MIC‡ (mg/L)	PK/PD non-species related breakpoint (mg/L)	Interpretation§
Gentamicin	1	0.5	Do not use
Ampicillin	≤0.016	2	Use with caution
Cefotaxime	0.004	1	Use with caution
Penicillin	≤0.016	0.25	Use with caution
Teicoplanin	0.032	No breakpoint	-
Vancomycin	0.25	No breakpoint	-
Clindamycin	1	No breakpoint	-
Erythromycin	0.032	No breakpoint	-
Linezolid	1	2	Use with caution
Ciprofloxacin	0.032	0.25	Use with caution
Moxifloxacin	0.016	0.25	Use with caution
Tetracycline	0.064	No breakpoint	-
Chloramphenicol	2	No breakpoint	-
Rifampin	0.004	No breakpoint	-

Globicatella sanguinis strain NCFB 2835 (American Type Culture Collection).
†*Globicatella sulfidifaciens* strain LMG 18844 (Belgian Coordinated Collections of Microorganisms), also known as DSM 15739.
‡MIC, MIC.
§Interpretation of susceptibility testing was based on PK/PD non-species related breakpoint, as recommended by EUCAST.

Reference

1. Letunic I, Bork P. Interactive Tree Of Life (iTOL) v5: an online tool for phylogenetic tree display and annotation. Nucleic Acids Res. 2021;49(W1):W293–6. PubMed https://doi.org/10.1093/nar/gkab301

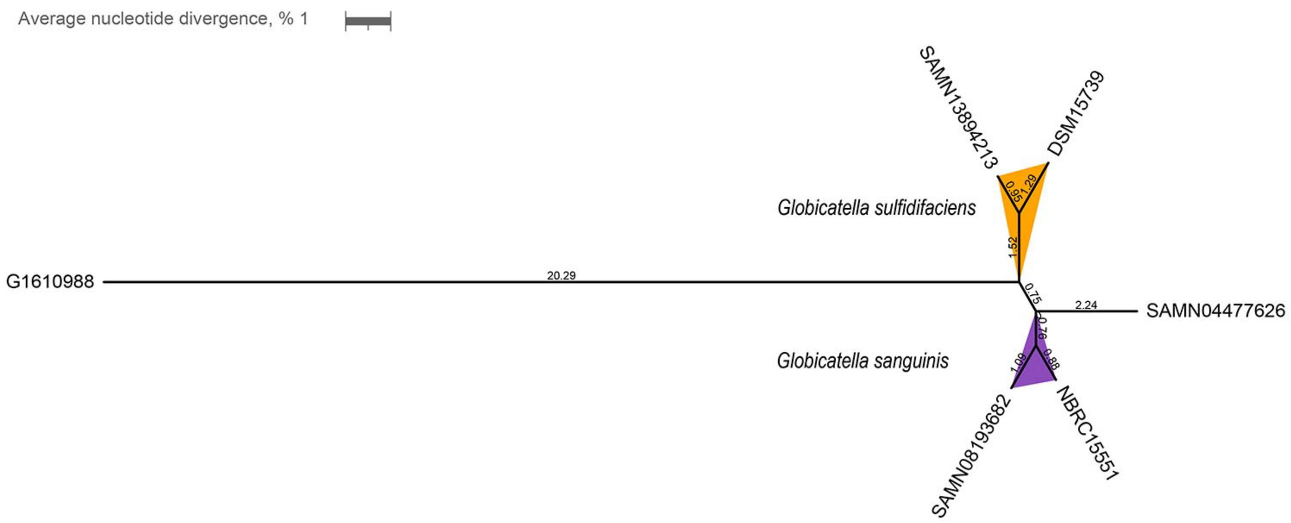

Appendix Figure. Average nucleotide divergence between isolate G1610988 and published *Globicatella* genomes. DSM15739 (NCBI Assembly accession: GCA_900167405.1) and NBRC15551 (GCA_001552295.1) are type strains of *G. sanguinis* and *G. sulfidifaciens*, respectively. Other published genomes: SAMN04477626 (GCA_001811625.1), SAMN08193682 (GCA_002847845.1), SAMN13894213 (GCA_012518335.1). Isolates of the same species are highlighted by colored shades. Numbers along branches are average percentage nucleotide divergencies of all orthologous regions shared between genomes. This figure was created by using iTOL (*1*).
